# Effects of respiratory muscle training on respiratory function, exercise capacity, and quality of life in chronic stroke patients: a systematic review and meta-analysis

**DOI:** 10.3389/fphys.2025.1642262

**Published:** 2025-09-18

**Authors:** Lang Huang, Jia-Mei Zhang, Zi-Ting Bi, Jing-Hua Xiao, Jing-Xue Wei, Jian Huang, Chao-Song Luo, Ying-Dong Li, Yue-Mi Zhang, Yun-Shan Zhang

**Affiliations:** ^1^ Department of Rehabilitation Medicine, The First Affiliated Hospital of Guangxi Medical University, Nanning, China; ^2^ Department of Rehabilitation Medicine, Guangxi International Zhuang Medicine Hospital, Nanning, China; ^3^ Cardiopulmonary Rehabilitation Center, Jiangbin Hospital of Guangxi Zhuang Autonomous Region, Nanning, China; ^4^ Department of Rehabilitation Medicine, The Guangxi Zhuang Autonomous Region Workers’ Hospital, Nanning, China

**Keywords:** chronic stroke, respiratory muscle training, respiratory function, exercise capacity, quality of life

## Abstract

**Background:**

Respiratory muscle training is a structured intervention targeting the respiratory muscles, yet its effect on chronic stroke patients remains unclear. The study evaluated the influence of this training on respiratory function, exercise capacity and quality of life among individuals who experienced chronic strokes.

**Methods:**

This study adhered to the PRISMA statement guidelines. A comprehensive search of databases including PubMed, Embase, AMED, CINAHL, Cochrane Library, and Web of Science was conducted without date limitations, extending until 8 March 2025. The search targeted randomised controlled trials that involved: 1) chronic stroke patients (≥18 years, diagnosed for >3 months), 2) respiratory muscle training encompasses both inspiratory and expiratory muscle training, and 3) outcomes measuring the strength and endurance of respiratory muscle, pulmonary function testing, exercise capacity, and quality of life. Two separate reviewers conducted the screening for eligibility, gathered data, and evaluated both the methodological quality and potential risk of bias. Meta-analyses utilized RevMan version 5.4 (Cochrane Collaboration, United Kingdom), applying random-effects models to calculate mean difference (MD), standardized mean difference (SMD), and corresponding 95% confidence intervals (95% CI).

**Results:**

Nine studies were included, comprising 288 participants (143 males and 145 females) with a mean age of 58.5 years. For primary outcomes, respiratory muscle training significantly enhanced maximal inspiratory pressure (MD = 17.71 cmH_2_O, 95% CI: 10.19–25.23) and respiratory muscle endurance (MD = 20.58 cmH_2_O, 95% CI: 12.25–28.92) among chronic stroke patients, but no significant effects were observed for maximal expiratory pressure (MD = 11.37 cmH_2_O, 95% CI: −0.78–25.23). The subgroup analysis revealed that the combination of inspiratory muscle training and expiratory muscle training enhanced maximal inspiratory pressure (MD = 23.47 cmH_2_O, 95% CI: 3.65–43.30) and respiratory muscle endurance (MD = 34.00 cmH_2_O, 95% CI: 21.21–46.79), while inspiratory muscle training improved maximal inspiratory pressure (MD = 14.09 cmH_2_O, 95% CI: 7.57–20.62), maximal expiratory pressure (MD = 8.69 cmH_2_O, 95% CI: 0.63–16.75), and respiratory muscle endurance (MD = 16.69 cmH_2_O, 95% CI: 10.27–23.11). For secondary outcomes, significant improvements occurred in forced expiratory volume in 1s (MD = 0.25 L, 95% CI: 0.06–0.44) and peak expiratory flow (MD = 0.84 L/s, 95% CI: 0.31–1.37), but not in forced vital capacity (MD = 0.16 L, 95% CI: −0.08–0.41), exercise capacity (SMD = 0.29, 95% CI: −0.03–0.61), and quality of life.

**Conclusion:**

Respiratory muscle training effectively enhances primary outcomes, including maximal inspiratory pressure and respiratory muscle endurance, as well as secondary outcomes such as forced expiratory volume in 1s and peak expiratory flow in chronic stroke patients, but does not improve maximal expiratory pressure, forced vital capacity, exercise capacity, and quality of life. The combination of inspiratory muscle training with expiratory muscle training, as well as inspiratory muscle training alone, can enhance maximal inspiratory pressure and the endurance of respiratory muscles. Furthermore, inspiratory muscle training alone can improve maximal expiratory pressure.

**Systematic Review Registration:**

identifier, CRD42024517859.

## 1 Introduction

Chronic stroke is a common neurological disease, usually caused by the consequences of acute stroke, leading to long-term functional impairment and disability, which significantly impacts the patients’ functional recovery and quality of life (Chan et al., 2022; Aguilera-Rubio et al., 2024; Briggs and O'Neill, 2016). Studies have pointed out that chronic stroke refers to the period more than 3 months after stroke onset, when the condition stabilizes and functional recovery reaches a relatively stable stage (Srivastava et al., 2010; Cameron et al., 2024). Chronic stroke patients often suffer from various functional disorders like motor dysfunction, cognitive dysfunction, and respiratory dysfunction, among which respiratory dysfunction is an important and common problem (Aguilera-Rubio et al., 2024; Kim et al., 2017; Li M. et al., 2024). Chronic stroke patients often experience varying degrees of respiratory dysfunction, primarily manifested by decreased respiratory muscle strength and endurance, reduced vital capacity, and decreased cough effectiveness (Deme et al., 2022). These impairments can easily lead to common problems such as decreased lung function, decreased activity tolerance, exertional dyspnea, and lung infections, making respiratory insufficiency a significant factor restricting the functional abilities and quality of life of this population (Deme et al., 2022). Currently, accurate reports on the incidence of respiratory dysfunction in chronic stroke are scarce, with research indicating that 30%–50% of these patients experience varying degrees of respiratory dysfunction (Kim et al., 2017; Patrizz et al., 2023; Jung et al., 2014). Individuals often focus on restoring respiratory dysfunction to enhance survival rates in acute stroke patients, while the issue is frequently overlooked in the chronic phase as priority is given to the recovery of motor function (Park, 2020; Xiao et al., 2012). Research has indicated that functional recovery in stroke patients occurs within 3 months of stroke onset, but can also continue to take place after 3 months or even years of stroke onset (Hebert et al., 2016; Langhorne et al., 2011). This indicates that the recovery of respiratory dysfunction plays a crucial role in the comprehensive functional rehabilitation of stroke patients who suffered from strokes at least 3 months, because impaired respiratory function directly affects key aspects like respiratory reserve, ventilation, oxygen delivery during exercise, and daily endurance and strength. Therefore, the research would concentrate on respiratory impairments in individuals who experienced strokes at least 3 months prior.

Stroke can damage the respiratory centre or corticospinal tract pathway, affect the activation of respiration and the activity of respiratory muscles, reduce the central respiratory drive and respiratory drive reserve, and impair the integration and regulation of respiratory-related sensory input (Billinger et al., 2012; Kim et al., 2014; Jo and Kim, 2017; Naghavi et al., 2019). For patients, this damage manifests as reduced cough efficiency, increased susceptibility to pneumonia, decreased respiratory reserve, inadequate ventilation, respiratory muscle fatigue, and decreased exercise tolerance, which together lead to respiratory dysfunction and an increased risk of respiratory failure (Spruit et al., 2013; Messaggi-Sartor et al., 2015; Wang et al., 2017; Teixeira-Salmela et al., 2005). For healthcare workers, these impairments require increased respiratory monitoring and intervention, increased healthcare workload and burden, and long-term use of healthcare resources to control recurrent infections and prevent exacerbations (Wang et al., 2017; Teixeira-Salmela et al., 2005). Studies have shown that chronic stroke respiratory dysfunction may be related to the existence of cortical-respiratory conduction disorders in chronic stroke patients (Liu et al., 2017; Vaz et al., 2021; Khedr et al., 2000; Urban et al., 2002). Besides, chronic stroke patients often experience decreased activity on the hemiplegic side, abnormal posture, and spasm of the chest wall muscle (Naghavi et al., 2019; Liu et al., 2017; Lima et al., 2014), which can lead to reduced lung volume, decreased chest wall compliance, and decreased coordination of respiratory muscle contraction, thus further worsening respiratory dysfunction (Martins et al., 2011; Lee et al., 2019). Respiratory dysfunction can increase the mortality rate of chronic stroke patients by 20%–40%, prolong hospitalization by one to 2 weeks, reduce cardiopulmonary fitness and endurance due to impaired inspiratory muscle coordination, reduced lung capacity and oxygen availability, and increased the load on respiratory muscles, aggravate motor dysfunction, and prolong recovery time (Finlayson et al., 2011; Hannawi et al., 2013; Rhoda et al., 2014). Chronic stroke patients with respiratory dysfunction are prone to fatigue when they need higher physical demands, especially intensive rehabilitation training, which can hinder recovery of exercise capacity, limit quality of life, and reduce the opportunity to live independently (Teixeira-Salmela et al., 2005; Hannawi et al., 2013). Thus, it is essential to investigate efficient respiratory rehabilitation strategies aimed at managing respiratory impairments in individuals who experienced chronic strokes, enhancing their overall functional recovery.

Respiratory muscle training (RMT) involves a therapeutic method that provides systematic and standardized training for respiratory muscles through handheld devices that provide pressure thresholds or flow-dependent resistance for inspiration or expiration, stimulate the respiratory muscles to respond and produce changes in muscle structure, improve maximum inspiratory pressure (MIP) and maximum expiratory pressure (MEP), thereby enhancing respiratory muscle strength and endurance, improving key respiratory functions including inspiratory capacity, ventilation function, breathing pattern efficiency, and cough peak flow, and increasing overall quality of life (Illi et al., 2012; Watson et al., 2022). The physiological rationale for RMT application is to induce physiological adaptations by enhancing neural drive to improve motor unit recruitment and synchronization, and promoting structural remodeling including diaphragm muscle fiber hypertrophy and a transition to a fatigue-resistant phenotype, while increasing mitochondrial density and capillarization through metabolic enhancement (Illi et al., 2012; van K et al., 2024). RMT encompasses strength-focused techniques including inspiratory muscle training (IMT) and expiratory muscle training (EMT), endurance-oriented approaches such as voluntary isocapnic hyperpnea (VIH) involving sustained hyperpnea with maintained CO_2_ levels, and hybrid modalities like tapered flow resistance devices that deliver variable loading throughout the respiratory cycle (Illi et al., 2012; Kowalski et al., 2024). While existing RMT studies in stroke predominantly focus on strength training (Fabero-Garrido et al., 2022; Menezes et al., 2016), VIH and tapered flow resistance training have been shown to be effective in healthy and active subjects (Illi et al., 2012; Kowalski et al., 2024). RMT has shown potential benefits in improving respiratory function, motor capacity and quality of life in multiple sclerosis, myasthenia gravis, and Parkinson’s disease (Ghannadi et al., 2022; Freitag et al., 2018; Zhuang and Jia, 2022). The findings suggest that RMT may have positive effects on functional recovery in neurological disorders by enhancing neuroplasticity through increased respiratory afferent input to the brainstem, improving cerebrovascular reactivity *via* CO_2_-mediated vasodilation, and optimizing oxygen delivery to neural tissues (Ghannadi et al., 2022; Zhuang and Jia, 2022). Additionally, RMT-induced enhancement of respiratory muscle strength and endurance can reduce respiratory oxygen consumption, potentially delaying muscle fatigue and redistributing blood flow from respiratory muscles to exercise muscles, leading to adaptive changes in respiratory muscles and thus redirecting cardiorespiratory resources to overall physical activity and recovery (Freitag et al., 2018; McConnell and Romer, 2004). If similar results are seen in patients with chronic stroke, it could provide a potential treatment. Preliminary clinical studies suggest that RMT may have the potential to improve respiratory muscle strength, exercise tolerance, and cardiorespiratory function after stroke (Sutbeyaz et al., 2010; Britto et al., 2011; Guillén-Solà et al., 2017). Nevertheless, the current evidence regarding the impact of RMT on patients with chronic stroke is still ambiguous. Consequently, it is essential to conduct a thorough review of existing clinical studies to assess the effects of RMT in this specific patient population.

Over the past few years, there has been a rise in the number of systematic reviews focusing on RMT for individuals who suffered from strokes. Nine systematic reviews (Fabero-Garrido et al., 2022; Menezes et al., 2016; Li L. et al., 2024; Zhang et al., 2024; Pozuelo-Carrascosa et al., 2020; Gomes-Neto et al., 2016; Menezes et al., 2018; Wu et al., 2020; Zhang et al., 2020) support that RMT can improve respiratory function in stroke patients, and one study (Pollock et al., 2013) suggests that RMT may improve respiratory function in stroke patients, but further research is needed. Seven systematic reviews (Fabero-Garrido et al., 2022; Menezes et al., 2016; Li L. et al., 2024; Zhang et al., 2024; Gomes-Neto et al., 2016; Menezes et al., 2018; Wu et al., 2020) found that RMT can promote exercise capacity in stroke patients. However, Xiao et al. (2012) presented an alternative perspective, arguing that the current evidence is inadequate to substantiate the impact of RMT on both respiratory function and exercise capacity in stroke patients. Only two studies (Xiao et al., 2012; Pozuelo-Carrascosa et al., 2020) investigated the effects of RMT on quality of life in stroke patients, but could not draw reliable conclusions. Unfortunately, these studies did not separately examine the impact of RMT on patients with chronic stroke. Only one meta-analysis focused on the impact of RMT on early-stage stroke patients, whereas other reviews incorporated findings from both acute and chronic stroke populations. Thus, considering the variations in recovery trajectories between individuals with acute and chronic strokes, it is necessary to conduct a systematic review and meta-analysis focusing on the efficacy of RMT in chronic stroke patients based on the current literature.

Hence, the study aimed to evaluate the impact of RMT on respiratory function, exercise capacity, and quality of life in chronic stroke patients.

## 2 Materials and methods

This systematic review and meta-analysis followed the preferred reporting items for systematic reviews and meta-analysis (PRISMA) guidelines (Page et al., 2021).

### 2.1 Eligibility criteria

The inclusion criteria were established (as outlined in Table 1) based on the Population-Intervention-Comparison-Outcome-Study Design (PICOS) framework. Notably, while cardiopulmonary exercise testing (CPET) is considered the gold standard for evaluating exercise capacity (Malhotra et al., 2016), the scarcity of CPET data in published studies investigating RMT for stroke survivors precluded its use as an inclusion criterion. Consequently, this review accepted standardized alternatives such as the 6-min walk test (6MWT) to ensure sufficient study inclusion and quantitative synthesis. The exclusion criteria were: (1) abstracts, letters, case reports, reviews, protocol, and unusable full text; (2) chronic stroke patients with congestive heart failure; (3) inappropriate intervention methods for unclear description of the training program about the intensity, duration, and frequency of the training; (4) studies not reporting the interesting outcome variables; (5) insufficient data for effect size (ES) and 95% confidence interval (CI); (6) studies of insufficient methodological quality with a PEDro score below 6 (Fabero-Garrido et al., 2022; Pozuelo-Carrascosa et al., 2020; Wood et al., 2008).

**TABLE 1:** Inclusion criteria.

**Population:** Chronic stroke patients aged ≥ 18 years and diagnosed for >3 months
**Intervention:** Respiratory muscle training encompasses both inspiratory and expiratory muscle training
**Control:** Placebo respiratory muscle training or rehabilitation program that does not incorporate respiratory muscle training
**Primary outcomes:** Maximal inspiratory pressure, maximal expiratory pressure, and respiratory muscle endurance
**Secondary outcomes:** Pulmonary function testing (peak expiratory flow, forced expiratory volume in 1s, forced vital capacity), exercise capacity (6-min walk test, maximum activity score), quality of life (euroquol 5-dimensions, nottingham health profile)
**Study Design**: Randomised Controlled Trials (RCTs)

### 2.2 Information sources

A comprehensive search was performed across various electronic databases including PubMed, Embase, AMED, CINAHL, Cochrane Library, and Web of Science without date limitations, extending up to 8 March 2025.

### 2.3 Search strategy

The keywords and associated mesh terms combined with Boolean operators and truncations were used to conduct comprehensive searches without language restrictions in the retrieval process to ensure that the retrieved outcomes were associated with the topic. The structured search strategies were formulated as follows. Following the main database search, the reference documents from all obtained studies were hand-examined to verify comprehensive resource identification. The detailed retrieval process for these databases can be found in Appendix 1.

(“stroke” OR “chronic stroke” OR “cerebrovascular accident” OR “CVA” OR “cerebral stroke” OR “brain vascular accident” OR “cerebrovascular stroke” OR “cerebrovascular apoplexy”) AND (“respiratory strength training” OR “inspiratory strength training” OR “expiratory strength training” OR “respiratory muscle training” OR “RMT” OR “inspiratory muscle training” OR “IMT” OR “expiratory muscle training” OR “EMT” OR “breathing muscle training” OR “breathing exercises”) AND (“respiratory function” OR “respiratory muscle strength” OR “maximum inspiratory pressure” OR “MIP” OR “maximum expiratory pressure” OR “MEP” OR “respiratory muscle endurance” OR “pulmonary function testing” OR “peak expiratory flow” OR “PEF” OR “forced expiratory volume in 1s” OR “FEV1” OR “forced vital capacity” OR “FVC” OR “exercise capacity” OR “6-min walk test” OR “6MWT” OR “maximum activity score” OR “MAS” OR “Quality of life” OR “euroquol 5-dimensions” OR “EQ5D” OR “nottingham health profile” OR “NHP”)

### 2.4 Selection process

The identified literature was imported into Endnote 20 for management, followed by the removal of duplicate entries. Subsequently, the researchers (LH and JM) independently evaluated titles and abstracts against predefined eligibility criteria during initial screening. Articles failing to meet inclusion requirements were systematically excluded before retrieving full-text documents for comprehensive quality assessment.

Full-text articles underwent systematic eligibility verification using pre-established selection parameters, with documented exclusion criteria for studies failing to meet inclusion criteria. Eventually, the reviewers (LH and JM) conducted joint validation for the final results. Inter-rater discrepancies were resolved through iterative deliberation with an independent reviewer (YS).

### 2.5 Data collection process

To minimize potential biases and errors, two independent investigators (YM and ZT) conducted data extraction from the included studies utilizing structured collection templates adapted from the Joanna Briggs Institute (JBI) tool. Since this tool is not only suitable for extracting data from various research designs including randomised controlled trial (RCT), but also can present the data clearly, making comparison and analysis easier (Aromataris et al., 2022). The extracted content encompassed: study identification (author/year/country), epidemiological constructs(design/sample size/participant profiles), interventional details (type of RMT/training load/frequency/length/device/supervision/progressive overload), outcome parameters, and other relevant information according to the suggestion of “Cochrane Handbook for Systematic Reviews of Interventions” (Cumpston et al., 2019). Any disagreements regarding data extraction content between the reviewers (YM and ZT) were mediated *via* consensual dialogue by an independent evaluator (JH). Additionally, the extracted content was verified by a third reviewer (JH). When some information was lacking, the investigator (JX) got in touch with the authors for acquiring.

### 2.6 Methodological quality and risk of bias assessment

The quality of retrieved studies can be identified through a critical assessment of methodological quality and the risk of bias, thus incorporating good quality research into the main review to ensure the effectiveness and rigour of the review itself (Morris et al., 2012; Tod et al., 2021). There are several tools to assess methodological quality and risk of bias, like the Cochrane Risk of Bias tool (Cumpston et al., 2019) and the PEDro score (Herbert et al., 1998). The methodological appraisal implemented the PEDro scale for quality assessment, with pre-specified evidence thresholds (≥6/10) systematically governing study eligibility determinations. Since the PEDro scale is not only an effective and reliable scoring tool for evaluating methodological quality but is also widely recognized in physical therapy (Herbert et al., 1998; Maher et al., 2003; Cashin and McAuley, 2020). The PEDro scale includes 11 items, including one external validity (eligibility criteria and source), eight items assessing the risk of bias (random allocation, concealed allocation, baseline comparability, blinding of participants, blinding of therapists, blinding of assessors, adequate follow-up (>85%), intention-to-treat analysis), and two items assessing the completeness of the statistical report on the risk of bias (between-group statistical comparisons, reporting of point measures and measures of variability) (Moseley et al., 2019). The total score ranges from 0 to 10 (the first item is not included), and higher scores indicate superior methodological quality (Maher et al., 2003). Studies with scores between 9 and 10 are considered ‘excellent’, and scores from six to 8 are assessed as good, whereas scores of 5 and 4 are classified as fair quality, and scores below 4 are considered poor quality (Foley et al., 2003; Gonzalez et al., 2018). Besides, the items of the Cochrane Risk of Bias tool were used to assess the risk of bias for the included studies, which were recorded in Review Manager 5.4 (Cochrane Collaboration, United Kingdom) (Cumpston et al., 2019). The Cochrane Risk of Bias tool contains six aspects of bias: selection bias, performance bias, detection bias, attrition bias, reporting bias, and other bias (Cumpston et al., 2019). Bias is evaluated as a judgment of various elements in the field (high, low, or unclear) (Cumpston et al., 2019). The methodological rigour and potential bias were independently appraised by two evaluators (JH and CS) through the PEDro criteria and the Cochrane Risk of Bias instruments. Any discrepancies in the assessment results were resolved by a third reviewer (YD).

### 2.7 Data synthesis and analysis

The outcome indicators were synthesized and analysed using RevMan 5.4 (Cochrane Collaboration, United Kingdom) (Cumpston et al., 2019). A meta-analysis was conducted solely when at least three RCTs were available for the variables studied (Fabero-Garrido et al., 2022). If meta-analysis was not available for each study outcome data, descriptive analysis would be performed. The heterogeneity among the studies was evaluated by the Cochrane Q statistic and the *I* ^2^ test (Cumpston et al., 2019). According to the Cochrane Handbook, an *I* ^2^ of 0%–40% is likely to be insignificant, 30%–60% may represent moderate heterogeneity, 50%–90% may indicate significant heterogeneity, and 75%–100% may illustrate considerable heterogeneity (Huedo-Medina et al., 2006). The study could be considered heterogeneous if the Cochran’s *Q* statistic tested significance (p < 0.1) or *I* ^2^ > 50% (Bown and Sutton, 2010). Due to concerns regarding the validity of the *Q* statistic in scenarios where the meta-analysis comprises a limited number of studies and exhibits substantial within-study variance (Fabero-Garrido et al., 2022), along with the unavoidable heterogeneity resulting from variations in participant demographics and intervention methods across the included studies, this research implemented a random effects model. Aggregated data for all findings were presented as either mean difference (MD) or standardized mean difference (SMD), alongside their corresponding 95% CI confidence intervals (95% CI). When the evaluation tools for outcome indicators were consistent, MD was used. Conversely, when the assessment tools were inconsistent, SMD was used. The difference was deemed significant at a test level of p < 0.05. When the mean and standard deviation (SD) were not available, and only the median along with the interquartile range (IQR) was provided, the CI estimates were adjusted by dividing the IQR by a factor of 1.35.

Subgroup analyses were conducted with an adequate number of studies to evaluate the impact of various forms of RMT, like IMT combined with EMT *versus* IMT alone. Additionally, a sensitivity analysis was carried out by sequentially excluding individual studies to examine the stability and dependability of the aggregated results pertaining to each variable. Furthermore, statistical assessment of publication bias through formal tests (e.g., Egger’s regression or Begg’s test) was not performed for any outcome, as the limited number of included studies (*k* < 10) renders such analyses statistically underpowered (Cumpston et al., 2019; Cumpston et al., 2022).

## 3 Results

**FIGURE 1:**
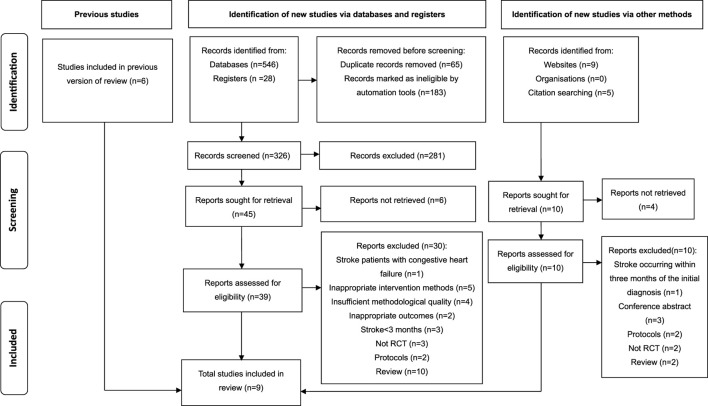
PRISMA search flow diagram

The comprehensive search yielded a total of 588 studies, comprising 546 articles sourced *via* electronic databases, 28 studies obtained from registries, and 14 studies identified through websites and citation searches. Subsequently, 248 studies with duplicates were removed, leaving 326 studies. Following the examination of titles and abstracts, a total of 281 articles deemed irrelevant were eliminated from consideration. Consequently, 45 studies remained, which necessitated the procurement and comprehensive review of their full texts, but the full texts of six studies could not be accessed. Simultaneously, 14 studies were retrieved from both website and citation searching, but 4 articles were not retrieved. Next, the 49 retrieved studies were assessed for eligibility. Finally, 9 studies (Vaz et al., 2021; Lee et al., 2019; Britto et al., 2011; Kim et al., 2011; Cho et al., 2018; Parreiras de Menezes et al., 2019; Liaw et al., 2020; Aydoğan et al., 2022; Zhu et al., 2024) met the eligibility criteria and were included for systematic review and meta-analysis, while the other 40 studies were excluded due to reasons like stroke occurring within 3 months of the diagnosis, inappropriate intervention methods and outcomes, and insufficient methodological quality. Figure 1 illustrates the PRISMA search flow diagram detailing the selection process for the studies.

### 3.1 Description of the included studies

The included nine studies were performed between 2011 and 2024, of which three studies (Vaz et al., 2021; Aydoğan et al., 2022; Zhu et al., 2024) were published in recent years to update the knowledge in this field and were not included in the previous review. The included studies varied by location, with three studies completed in Korea (Lee et al., 2019; Kim et al., 2011; Cho et al., 2018) and in Brazil (Vaz et al., 2021; Britto et al., 2011; Parreiras de Menezes et al., 2019) respectively, two in China (Liaw et al., 2020; Zhu et al., 2024), and one in Turkey (Aydoğan et al., 2022). Although these studies are geographically limited and cannot represent the entire global trend, the integrated evidence may provide reference and guidance for future clinical practice guidance and study. Table 2 presents a summary of the features of the 9 studies.

**TABLE 2:** Characteristics of the included nine studies.

Study	Country	Participants/Mean age	Intervention	Control	Outcome measures	Main results
Zhu et al. (2024)	China	n = 50 IG: 25 CG: 25 Male/female: 40/10 62.8 years Stroke >6 months MIP: 50.5 cmH_2_O	Type of RMT: IMT Training load: 30% of MIP Frequency: 2 sets of 30 breaths, once a day, 6 days/week Length: 4 weeks Device: electronic inspiratory loading device (threshold resistance) (POWERbreathe K5, HaB International Ltd., United Kingdom) Supervision: supervised by the trainer Progressive overload: NR Regular rehabilitation program as CG	Regular rehabilitation program: external diaphragmatic pacing therapy (20 min per day, 6 days/week, 4 weeks)	Respiratory function: MIP, FVC, FEV1 Exercise capacity: N/A Quality of life: N/A	There were significant between-group differences for MIP, FVC, and FEV1
Aydoğan et al. (2022)	Turkey	n = 21 IG: 11 CG: 10 Male/female: 7/14 63.9 years Stroke >3 months MIP: 59.7 cmH_2_O MEP: 73.4 cmH_2_O	Type of RMT: IMT Training load: 40% of MIP Frequency: 15 min in 2 sessions, 30 min-per day, 7 days/week Length: 6 weeks Device: pressure threshold-loading device (threshold resistance) (Philips Respironics, Pittsburgh, PA, United States) Supervision: supervised by the trainer Progressive overload: resistance adjusted by the repeated measurements each week Regular rehabilitation program as CG	Regular rehabilitation program: neurodevelopmental treatment (5 days/week, 6 weeks)	Respiratory function: MIP, MEP, FVC, FEV1, PEF Exercise capacity: 6MWT Quality of life: N/A	Significant differences were observed between both groups in MIP and PEF, but no significant difference in MEP, FVC, FEV1 and 6MWT
Vaz et al. (2021)	Brazil	n = 50 IG: 23 CG: 27 Male/female: 21/29 54 years 6 months < Stroke <5 years MIP: 47 cmH_2_O MEP: 66 cmH_2_O	Type of RMT: IMT Training load: 50% of MIP Frequency: 30 min/day (two sets of 15 min/day), 5 times/week Length: 6 weeks Device: POWERbreathe Medic Plus equipment (threshold resistance) Supervision: supervised by the trainer Progressive overload: resistance adjusted weekly Regular rehabilitation program as CG	Regular rehabilitation program: upper extremity function, static and dynamic balance, gait training in different terrains, mobility and transfers, joint mobilization exercises, and muscle flexibility (seven to eight 1-h sessions per week, 6 weeks) Sham respiratory muscle training at minimum load (1 cmH_2_O)	Respiratory function: MIP, MEP, RME Exercise capacity: 6MWT Quality of life: EQ5D	There were significant between-group differences for RME There was no significant between-group difference for MIP, MEP, 6MWTEQ5D
Liaw et al. (2020)	China	n = 31 IG: 15 CG: 16 Male/female: 12/19 62.8 years Stroke >6 months MIP: 45.9 cmH_2_O MEP: 50.1 cmH_2_O	Type of RMT: IMT + EMT Training load: 30%–60% of MIP and15%–75% of MEP Frequency: 5 repetitions, 1 to 2 times per day, 5 days/week Length: 6 weeks Device: the Dofin Breathing Trainer (DT 11 orDT 14 GaleMed Corporation) Supervision: supervised by the researcher Progressive overload: resistance adjusted weekly by the participant’s tolerance Regular rehabilitation program as CG	Regular rehabilitation program: postural training, breathing control, improving cough technique, checking chest wall mobility, fatigue management, orofacial exercises, thermal tactile stimulation, mendelsohn manoeuvering, effort swallowing, or supra-glottic manoeuver among others	Respiratory function: MIP, MEP, FVC, FEV1 Exercise capacity: N/A Quality of life: N/A	Significant differences were observed between both groups in MIP, FVC, and FEV1, but no significant difference in MEP
Lee et al. (2019)	Korea	n = 25 IG: 13 CG: 12 Male/female: 12/13 59.1 years Stroke >6 months MIP: 33.5 cmH_2_O MEP: 43.2 cmH_2_O	Type of RMT: IMT + EMT Training load: 30% of MEP and MIP Frequency: repeated 10–15 times, 20 min/day, 3 times/week Length: 6 weeks Device: handheld expiratory/inspiratory-type pressure threshold devices (variable resistance) (Threshold positive expiratory pressure, Threshold IMT-Philips Respironics, Andover, MA) Supervision: supervised by the trainer Progressive overload: resistance adjusted according to the patient’s ability weekly Regular rehabilitation program as CG	Regular rehabilitation program: conventional physical and occupational therapy (30 min, 2 times a day, 6 times per week, 6 weeks) Trunk stabilization exercises (20 min, 3 times a week, 6 weeks)	Respiratory function: MIP, MEP, FEV1, PEF Exercise capacity: N/A Quality of life: N/A	There were significant differences between the two groups in MIP, MEP and PEF FEV1 showed no significant differences between the two groups
Parreiras de Menezes et al. (2019)	Brazil	n = 38 IG: 19 CG: 19 Male/female: 16/22 63.5 years 3 months < stroke <5 years MIP: 55 cmH_2_O MEP: 76 cmH_2_O	Type of RMT: IMT + EMT Training load: 50% of MEP and MIP Frequency: two 20-min sessions (morning and afternoon), 40 min/day, 7 times/week Length: 8 weeks Device: Orygen-dual valve (variable resistance) (Orygen-dual valve; Forumed S.L) Supervision: supervised by the trainer Progressive overload: resistance adjusted weekly according to the current load value of measuring the inspiratory and expiratory strength	Sham respiratory training using the same device, without any resistance or progression	Respiratory function: MIP, MEP, RME Exercise capacity: 6MWT Quality of life: N/A	There were significant between-group differences for MIP, MEP and RME There was no significant between-group difference for 6MWT
Cho et al. (2018)	Korea	n = 25 IG: 12 CG: 13 Male/female: 13/12 49.5 years Stroke >3 months MIP: 55 cmH_2_O	Type of RMT: IMT Training load: 30% of MIP Frequency: 3 sets of 30 breaths, 5 days/week Length: 6 weeks Device: POWERbreathe K5 (variable resistance) (HaB International Ltd., United Kingdom) Supervision: supervised by the trainer Progressive overload: resistance readjusted weekly Regular rehabilitation program as CG	Regular rehabilitation program: muscle strengthening exercises, therapy using the bobath approach, general gait training, and stair climbing training (60 min a day, 5 days/week, 6 weeks)	Respiratory function: MIP, RME Exercise capacity: 6MWT Quality of life: N/A	There were significant differences between the two groups in MIP and RME There was no significant between-group difference for 6MWT
Britto et al. (2011)	Brazil	n = 21 IG: 11 CG: 10 Male/female: 12/9 54 years Stroke >9 months MIP: 56.7 cmH_2_O	Type of RMT: IMT Training load: 30% of MIP Frequency: 30 min/day, 5 times/week Length: 8 weeks Device: Threshold IMT (threshold resistance) Supervision: supervised by the researcher Progressive overload: resistance readjusted biweekly	Sham respiratory muscle training without the threshold resistance valve (30 min/day, 5 times/week, 8 weeks)	Respiratory function: MIP, RME Exercise capacity: MAS Quality of life: NHP	There were significant between-group differences for MIP and RME No statistically significant differences were observed for MAS and NHP between groups
Kim et al. (2011)	Korea	n = 27 IG: 13 C: 14 Male/female:10/17 57 years Stroke >6 months MIP: NR MEP: NR	Type of RMT: IMT + EMT Training load: 50%–60% of the subject’s VC Frequency: 30 min/day, 3 days/week Length: 4 weeks Device: The SpiroTiger (flow resistance) Supervision: supervised by a researcher Progressive overload: NR Regular rehabilitation program as CG	Regular rehabilitation program: conventional physical therapy (30 min/day, 3 days/week, 4 weeks)	Respiratory function: FVC, FEV1, PEF Exercise capacity: N/A Quality of life: N/A	There were significant between-group differences for FVC, FEV1, and PEF

Abbreviations: IG, Intervention group; CG, Control group; RMT, Respiratory muscle training; IMT, Inspiratory muscle training; EMT, Expiratory muscle training; MIP, Maximal inspiratory pressure; MEP, Maximal expiratory pressure; RME, Respiratory muscle endurance; VC, Vital capacity; FVC, Forced vital capacity; FEV1, Forced expiratory volume in 1s; PEF, peak expiratory flow; 6MWT, Six-minute walk test; MAS, Maximum activity score; EQ5D, euroquol 5-dimensions; NHP, Nottingham health profile; NR, not reported in the source trial; N/A, Not available.

#### 3.1.1 Participants

A total of 288 participants were enrolled in these studies, with the number of participants ranging from 21 (Britto et al., 2011; Aydoğan et al., 2022) to 50 (Vaz et al., 2021; Zhu et al., 2024). The mean age of individuals was 58.5 years. Participants in each study included both males and females, totalling 143 males and 145 females. All study participants suffered a stroke more than 3 months prior to the diagnosis. Five studies (Vaz et al., 2021; Lee et al., 2019; Kim et al., 2011; Liaw et al., 2020; Zhu et al., 2024) included patients with more than 6 months of stroke. One study included patients with longer than 9 months of stroke (Britto et al., 2011). Others included patients with over 3 months of stroke (Cho et al., 2018; Parreiras de Menezes et al., 2019; Aydoğan et al., 2022). Additionally, all studies evaluated the initial MIP and MEP separately except for the study by Kim et al. (2011), where five studies (Vaz et al., 2021; Lee et al., 2019; Parreiras de Menezes et al., 2019; Liaw et al., 2020; Aydoğan et al., 2022) assessed the initial MIP and MEP, while the remaining three studies (Britto et al., 2011; Cho et al., 2018; Zhu et al., 2024) evaluated MIP. The mean values for the original MIP and MEP were 50.4 cmH_2_O and 61.7 cmH_2_O respectively. Reference values for healthy adults show MIP and MEP as follows: MIP (118.4 ± 37.2 cmH_2_O for males and 84.5 ± 30.3 cmH_2_O for females) and MEP (140 ± 30 cmH_2_O for males and 95 ± 20 cmH_2_O for females) (Evans and Whitelaw, 2009; Gil et al., 2012). These respiratory muscle strength reference values are from Manizales (altitude of 2150 m asl) (Evans and Whitelaw, 2009; Gil et al., 2012), so they may be not universal for sea-level residents and sea-level born. If the values are lower than normal, it is considered a decrease in MIP and MEP (Evans and Whitelaw, 2009; Gil et al., 2012). Thus, the initial average values of MIP and MEP reported in this study are interpreted as a decline.

#### 3.1.2 Interventions

All included studies conducted RMT in the intervention group (IG), five studies (Vaz et al., 2021; Britto et al., 2011; Cho et al., 2018; Aydoğan et al., 2022; Zhu et al., 2024) only had IMT, and the remaining four studies (Lee et al., 2019; Kim et al., 2011; Parreiras de Menezes et al., 2019; Liaw et al., 2020) were IMT and EMT. In the control group (CG), most studies (Lee et al., 2019; Kim et al., 2011; Cho et al., 2018; Liaw et al., 2020; Aydoğan et al., 2022; Zhu et al., 2024) carried out regular stroke rehabilitation programs, which also were performed in the IG. Interestingly, two studies (Britto et al., 2011; Parreiras de Menezes et al., 2019) used sham RMT without any resistance, and one study (Vaz et al., 2021) performed sham respiratory muscle training at a minimum load (1 cmH_2_O) that was not enough to change respiratory muscle strength or endurance. Regarding the use of devices in the IG, two studies adopted POWERbreathe Medic Plus (Vaz et al., 2021; Liaw et al., 2020), while the others were different: electronic inspiratory loading device (Zhu et al., 2024), the SpiroTiger (Kim et al., 2011), POWERbreathe K5 (Cho et al., 2018), Orygen-dual valve (Parreiras de Menezes et al., 2019), Dofin Breathing Trainer (Liaw et al., 2020), handheld expiratory/inspiratory-type pressure threshold devices (Lee et al., 2019), Threshold IMT (Britto et al., 2011). Six studies (Lee et al., 2019; Cho et al., 2018; Parreiras de Menezes et al., 2019; Liaw et al., 2020; Aydoğan et al., 2022; Zhu et al., 2024) reported the manufacturers (Details in Table 2), while the remaining three studies (Vaz et al., 2021; Britto et al., 2011; Kim et al., 2011) did not report the manufacturers. The majority of studies (Vaz et al., 2021; Britto et al., 2011; Parreiras de Menezes et al., 2019; Liaw et al., 2020; Aydoğan et al., 2022; Zhu et al., 2024) used threshold resistance, Cho et al. (2018) and Lee et al. (2019) used variable resistance, and only Kim et al. (2011) mentioned flow resistance, despite the use of various device types.

The parameters of RMT varied among the included studies. The majority of studies investigating RMT commenced at a training load ranging from 30% to 60% of MIP and 15%–75% of MEP and was readjusted with the intervention weekly or biweekly according to different situations like the repeated measurements, the participant’s tolerance or ability, the current load value of measuring the inspiratory and expiratory strength. However, only Kim et al. (2011) implemented a training load of 50%–60% of VC alongside a low frequency of 12–13 breaths per minute, which was different from the indicators used in others. Despite the initial training load of RMT differing among the included studies, it is important to highlight that most studies maintained a training load exceeding 30% of MIP. MIP with a load of less than 30% may not be sufficient to improve inspiratory muscle strength and exercise tolerance (Fabero-Garrido et al., 2022). The frequency and length of RMT were different across the studies. The time of frequency varied from 20 to 40 min, and four studies (Vaz et al., 2021; Britto et al., 2011; Kim et al., 2011; Aydoğan et al., 2022) adopted 30 min. Four studies (Lee et al., 2019; Cho et al., 2018; Liaw et al., 2020; Zhu et al., 2024) used 2 or 3 sets with 5, 10–15 or 30 repetitions. Sessions were conducted 3 to 7 times every week. Furthermore, the length of RMT was observed to span from 4 to 8 weeks. Most studies (Vaz et al., 2021; Lee et al., 2019; Britto et al., 2011; Cho et al., 2018; Parreiras de Menezes et al., 2019; Liaw et al., 2020; Aydoğan et al., 2022) tended to 6 weeks. All study interventions were conducted under supervision.

### 3.2 Methodological quality and risk of bias of the studies

**FIGURE 2:**
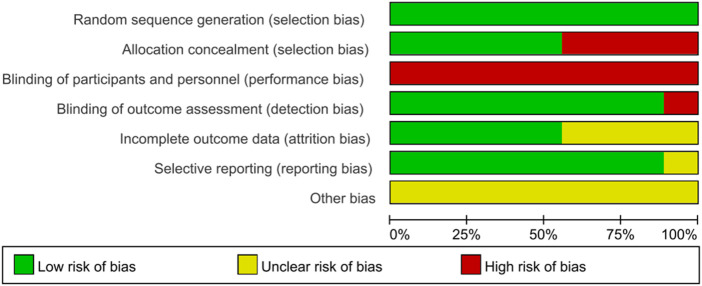
Risk of bias graph for the studies.

The methodological quality and potential biases of the studies included in this review were meticulously evaluated using the PEDro scale and the Cochrane Risk of Bias tool. Table 3 summarizes the total results and item scores of the PEDro scale for the included 9 RCTs. Moreover, the risk of bias graph relevant to these studies is displayed in Figure 2. Notably, the investigation conducted by Parreiras de Menezes et al. (2019) achieved the highest score, obtaining 8 points on the PEDro scale, thereby categorizing its methodological quality as ‘good’. The remaining eight studies (Vaz et al., 2021; Lee et al., 2019; Britto et al., 2011; Kim et al., 2011; Cho et al., 2018; Liaw et al., 2020; Aydoğan et al., 2022; Zhu et al., 2024) were considered to be of good methodological quality with a PEDro score of six points or seven points. The average PEDro score across all included studies was 6.7, with scores ranging from 6 to 8, indicating that the body of evidence is of good quality.

**TABLE 3:** Study quality on the PEDro Scale of nine studies.

Study	Random Allocation	Concealed Allocation	Baseline Similarity	Blind subjects	Blind therapists	Blind assessors	Adequate follow-up	Intention-to-treat analysis	Between-group comparisons	Point estimates and variability	Total Score
Zhu et al. (2024)	Y	Y	Y	N	N	Y	Y	N	Y	Y	7
Aydoğan et al. (2022)	Y	N	Y	N	N	Y	Y	Y	Y	Y	7
Vaz et al. (2021)	Y	N	Y	Y	N	Y	N	Y	Y	Y	7
Liaw et al. (2020)	Y	N	Y	N	N	Y	N	Y	Y	Y	6
Lee et al. (2019)	Y	Y	Y	N	N	Y	N	N	Y	Y	6
Parreiras de Menezes et al. (2019)	Y	Y	Y	Y	N	N	Y	Y	Y	Y	8
Cho et al. (2018)	Y	Y	Y	N	N	Y	N	N	Y	Y	6
Britto et al. (2011)	Y	Y	Y	N	N	Y	Y	N	Y	Y	7
Kim et al. (2011)	Y	N	Y	Y	N	Y	N	N	Y	Y	6

The total score of PEDro: 10.

Abbreviations: PEDro, Physiotherapy Evidence Database; Y, Yes; N, No.

Each of the rigorously evaluated studies offered insights into random allocation, baseline comparability, inter-group differences, as well as point estimates and their variability. Eight studies (Vaz et al., 2021; Lee et al., 2019; Britto et al., 2011; Kim et al., 2011; Cho et al., 2018; Liaw et al., 2020; Aydoğan et al., 2022; Zhu et al., 2024) conducted blind assessors, except for the study by Parreiras de Menezes et al. (2019). Five studies (Lee et al., 2019; Britto et al., 2011; Cho et al., 2018; Parreiras de Menezes et al., 2019; Zhu et al., 2024) reported concealed allocation, whereas the other four studies (Vaz et al., 2021; Kim et al., 2011; Liaw et al., 2020; Aydoğan et al., 2022) did not implement this method, which may introduce selection bias. Four studies provided respectively intention-to-threat analysis (Vaz et al., 2021; Parreiras de Menezes et al., 2019; Liaw et al., 2020; Aydoğan et al., 2022) and adequate follow-up (Britto et al., 2011; Parreiras de Menezes et al., 2019; Aydoğan et al., 2022; Zhu et al., 2024). Unfortunately, only three studies (Vaz et al., 2021; Kim et al., 2011; Parreiras de Menezes et al., 2019) involved blind subjects, and none included blind therapists. Neither participants nor therapists were blinded, which may potentially lead to performance bias. Notably, performance bias should not be considered a form of preferential bias, as it is often challenging or unfeasible to blind participants and therapists when implementing complex interventions (Rodrigues-Baroni et al., 2014). Thus, although there were performance biases in these studies, this study accepted the factor and considered it when interpreting the findings.

### 3.3 Effect of interventions

#### 3.3.1 Effect of respiratory muscle training on respiratory muscle strength

**FIGURE 3:**
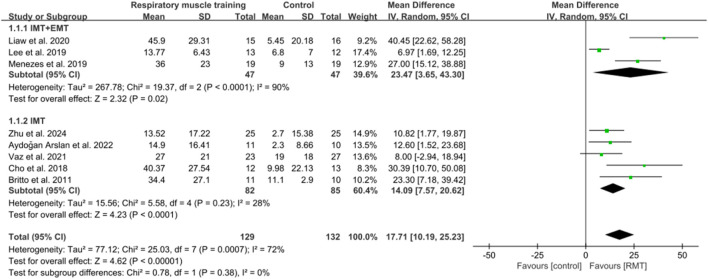
Forest plot showing the pooled effect size of RMT on MIP across RMT and control groups.

**FIGURE 4:**
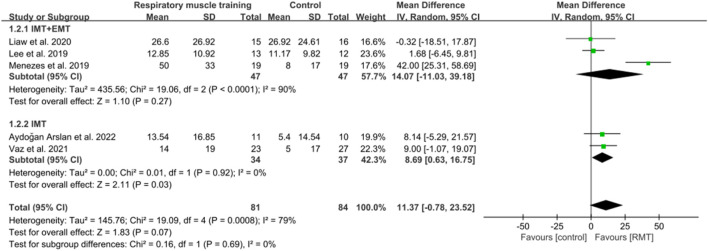
Forest plot showing the pooled effect size of RMT on MEP across RMT and control groups.

Eight studies (Vaz et al., 2021; Lee et al., 2019; Britto et al., 2011; Cho et al., 2018; Parreiras de Menezes et al., 2019; Liaw et al., 2020; Aydoğan et al., 2022; Zhu et al., 2024) showed results about MIP in units of cmH_2_O, while five studies (Vaz et al., 2021; Lee et al., 2019; Parreiras de Menezes et al., 2019; Liaw et al., 2020; Aydoğan et al., 2022) analysed MEP in units of cmH_2_O. The aggregated data indicated that RMT significantly enhanced MIP among chronic stroke patients compared to the CG (n = 261, MD = 17.71, 95% CI: 10.19–25.23, p < 0.00001, *I* ^2^ = 72%; Figure 3), exceeding the minimal clinically important difference (MCID) threshold of 9 cmH_2_O for respiratory weakness (Menezes et al., 2016). However, RMT did not show any significant effect on increasing MEP among chronic stroke patients (n = 165, MD = 11.37, 95% CI: −0.78 to 25.23, p = 0.07, *I* ^2^ = 79%; Figure 4). The sensitivity exclusion analysis indicated that none of the studies significantly changed the pooled results for MIP and MEP when analysed individually. The leave-one-out sensitivity analyses for MIP and MEP.

Three studies (Lee et al., 2019; Parreiras de Menezes et al., 2019; Liaw et al., 2020) carried out both IMT and EMT for MIP and MEP. Five studies (Vaz et al., 2021; Britto et al., 2011; Cho et al., 2018; Aydoğan et al., 2022; Zhu et al., 2024) only performed IMT for MIP, while two studies (Vaz et al., 2021; Aydoğan et al., 2022) only carried out IMT for MEP. IMT + EMT yielded a notable enhancement in MIP (n = 94, MD = 23.47, 95% CI: 3.65–43.30, p = 0.02, *I* ^2^ = 90%; Figure 3), but no evidence of effect could be found for IMT + EMT on increasing MEP (n = 94, MD = 14.07, 95% CI: −11.03–39.18, p = 0.27, *I* ^2^ = 90%; Figure 4). Sensitivity analysis regarding study exclusions revealed that none of the studies significantly affected the pooled result of MEP. However, after excluding the study of Liaw et al. (2020) and Parreiras de Menezes et al. (2019), IMT + EMT did not yield a notable enhancement in MIP. Besides, IMT demonstrated a significant enhancement in MIP (n = 167, MD = 14.09, 95% CI: 7.57–20.62, p < 0.0001, *I* ^2^ = 28%; Figure 3) and MEP (n = 71, MD = 8.69, 95% CI: 0.63–16.75, p = 0.03, *I* ^2^ = 0%; Figure 4). Sensitivity analysis regarding study exclusions showed that none of the studies significantly influenced the result of MIP. However, after excluding the studies by Vaz et al. (2021) and Aydoğan et al. (2022) separately, no evidence was found that IMT affected improving MEP. The leave-one-out sensitivity analyses for MIP and MEP pertaining to subgroup analyses are presented. Notably, there was no significant difference observed between the effects of IMT + EMT and IMT alone on MIP (p = 0.38; Figure 3) and MEP (p = 0.69; Figure 4).

#### 3.3.2 Effect of respiratory muscle training on respiratory muscle endurance

**FIGURE 5:**
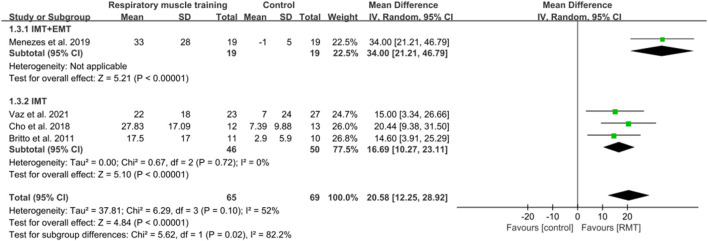
Forest plot showing the pooled effect size of RMT on respiratory muscle endurance across RMT and control groups.

Four (Vaz et al., 2021; Britto et al., 2011; Cho et al., 2018; Parreiras de Menezes et al., 2019) studies measured the results of respiratory muscle endurance in units of cmH_2_O. The aggregated findings suggested that RMT significantly improved respiratory muscle endurance in chronic stroke patients when compared to the CG. (n = 134, MD = 20.58, 95% CI: 12.25–28.92, p < 0.00001, *I* ^2^ = 52%; Figure 5). The sensitivity analysis indicated that no study significantly influenced the pooled results concerning respiratory muscle endurance. The leave-one-out sensitivity analyses for respiratory muscle endurance.

One study (Parreiras de Menezes et al., 2019) conducted both IMT and EMT, while three studies (Vaz et al., 2021; Britto et al., 2011; Cho et al., 2018) only performed IMT. IMT + EMT and IMT resulted in a significant increase in respiratory muscle endurance (n = 38, MD = 34.00, 95% CI: 21.21–46.79, p < 0.00001; n = 96, MD = 16.69, 95% CI: 10.27–23.11, p < 0.00001, *I* ^2^ = 0%; Figure 5). The sensitive exclusion analysis indicated that no study significantly changed this result for IMT. Due to the existence of only one study, a sensitivity analysis could not be performed for IMT + EMT. The leave-one-out sensitivity analyses for respiratory muscle endurance pertaining to subgroup analyses are presented. A statistically significant difference was observed between the effects of IMT + EMT and IMT alone in enhancing respiratory muscle endurance (p = 0.02; Figure 5).

#### 3.3.3 Effect of respiratory muscle training on pulmonary function

**FIGURE 6:**
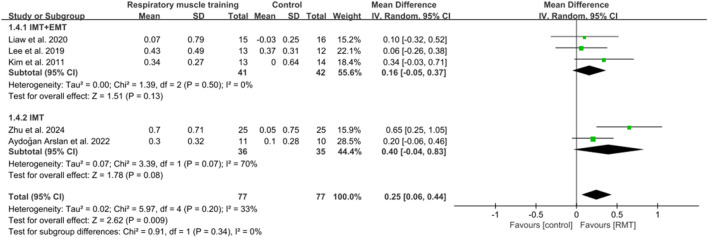
Forest plot showing the pooled effect size of RMT on FEV1 across RMT and control groups.

**FIGURE 7:**
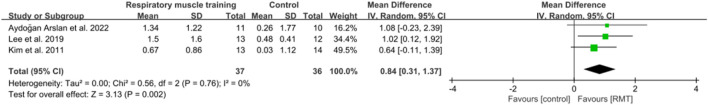
Forest plot showing the pooled effect size of RMT on PEF across RMT and control groups.

**FIGURE 8:**
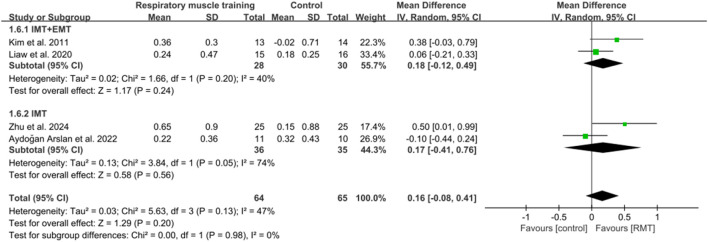
Forest plot showing the pooled effect size of RMT on FVC across RMT and control groups.

Five studies (Lee et al., 2019; Kim et al., 2011; Liaw et al., 2020; Aydoğan et al., 2022; Zhu et al., 2024) reported the results on pulmonary function. These studies all analysed forced expiratory volume in 1s (FEV1) in units of L, while three studies (Lee et al., 2019; Kim et al., 2011; Aydoğan et al., 2022) showed peak expiratory flow (PEF) in units of L/s and four studies (Kim et al., 2011; Liaw et al., 2020; Aydoğan et al., 2022; Zhu et al., 2024) showed results regarding forced vital capacity (FVC) in units of L. The pooled data suggested that RMT had a statistically significant influence on FEV1 (n = 154, MD = 0.25, 95% CI: 0.06–0.44, p = 0.009, *I* ^2^ = 33%; Figure 6) and PEF (n = 73, MD = 0.84, 95% CI: 0.31–1.37, p = 0.002, *I* ^2^ = 0%; Figure 7) in patients with chronic stroke compared to the CG. The improvement in FEV1 reached the lower limit of MCID for airway obstruction (0.25 L) (Chen et al., 2025), while the improvement in PEF exceeded the MCID of 0.5 L/s for cough efficacy (Zhang et al., 2022). Unfortunately, no evidence of effect could be found for RMT on increasing FVC compared to the CG (n = 129, MD = 0.16, 95% CI: −0.08–0.41, p = 0.2, *I* ^2^ = 47%; Figure 8). Sensitivity analysis regarding study exclusions indicated that the pooled results of PEF and FVC remained unchanged when each study was excluded. Notably, RMT did not achieve a statistically significant improvement in FEV1 after excluding the study by Kim et al. (2011). The leave-one-out sensitivity analyses of FEV1, PEF, and FVC.

In terms of FEV1, three studies (Lee et al., 2019; Kim et al., 2011; Liaw et al., 2020) performed both IMT and EMT, while two studies (Aydoğan et al., 2022; Zhu et al., 2024) only carried out IMT. Regarding FVC, two studies (Kim et al., 2011; Liaw et al., 2020) conducted both IMT and EMT, and other studies (Aydoğan et al., 2022; Zhu et al., 2024) did only IMT. No evidence of effect could be found for IMT + EMT and IMT on improving FEV1 (n = 83, MD = 0.16, 95% CI: −0.05–0.37, p = 0.13, *I* ^2^ = 0%; n = 71, MD = 0.4, 95% CI: −0.04–0.83, p = 0.08, *I* ^2^ = 70%; Figure 6) and FVC (n = 58, MD = 0.18, 95% CI: −0.12–0.49, p = 0.24, *I* ^2^ = 40%; n = 71, MD = 0.17, 95% CI: −0.41–0.76, p = 0.56, *I* ^2^ = 74%; Figure 8). Sensitivity analysis regarding study exclusions indicated that no study changed the results of FEV1 and FVC for IMT + EMT. Interestingly, after excluding the study of Aydoğan et al. (2022), IMT produced a statistically significant improvement in FEV1 and FVC. The leave-one-out sensitivity analyses of FEV1 and FVC pertaining to subgroup analyses. There was no significant difference observed between the effects of IMT + EMT and IMT alone on FEV1 (p = 0.34; Figure 6) and FVC (p = 0.98; Figure 8). Since only three studies separately evaluated PEF, subgroup analysis of IMT + EMT and IMT could not be performed.

#### 3.3.4 Effect of respiratory muscle training on exercise capacity

**FIGURE 9:**
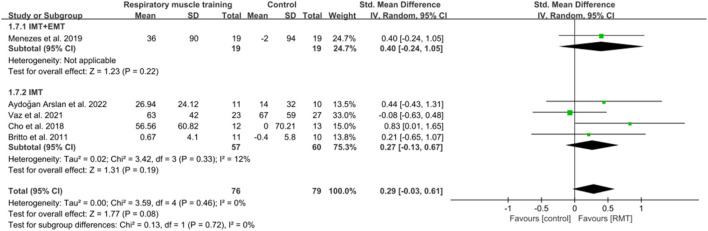
Forest plot showing the pooled effect size of RMT on exercise capacity across RMT and control groups.

Five studies (Vaz et al., 2021; Britto et al., 2011; Cho et al., 2018; Parreiras de Menezes et al., 2019; Aydoğan et al., 2022) collected exercise capacity data. Four studies (Vaz et al., 2021; Cho et al., 2018; Parreiras de Menezes et al., 2019; Aydoğan et al., 2022) evaluated the walking ability of patients with chronic stroke by measuring 6MWT (m), while one study (Britto et al., 2011) assessed MAS. The meta-analysis was performed utilizing SMD owing to discrepancies in the evaluation scales. The pooled data suggested that no evidence of effect could be found for RMT on improving exercise capacity among chronic stroke patients compared to the CG (n = 155, SMD = 0.29, 95% CI: −0.03–0.61, p = 0.08, *I* ^2^ = 0%; Figure 9). However, the sensitivity analysis showed that RMT produced a statistically significant improvement in exercise capacity after excluding the study by Vaz et al. (2021). The leave-one-out sensitivity analyses for exercise capacity.

Only one study (Parreiras de Menezes et al., 2019) performed both IMT and EMT, while four studies (Vaz et al., 2021; Britto et al., 2011; Cho et al., 2018; Aydoğan et al., 2022) carried out only IMT. No evidence of effect could be found for IMT + EMT and IMT on changing exercise capacity (n = 38, SMD = 0.4, 95% CI: −0.24–1.05, p = 0.22; n = 117, SMD = 0.27, 95% CI: −0.13–0.67, p = 0.19, *I* ^2^ = 12%; Figure 9). The sensitive exclusion analysis showed that IMT achieved a statistically significant improvement in exercise capacity when the study of Vaz et al. (2021) was removed. The leave-one-out sensitivity analyses for exercise capacity pertaining to subgroup analyses. It was not possible to perform a sensitivity analysis concerning exercise capacity in IMT + EMT, as only a single study was accessible. No statistically significant difference was identified in the enhancement of exercise capacity between IMT + EMT and IMT (p = 0.72; Figure 9).

#### 3.3.5 Effect of respiratory muscle training on quality of life

Only two studies showed quality of life by using NHP scores (Britto et al., 2011) and EuroQol5D scales (Vaz et al., 2021) separately. Due to the scarcity of research focused on quality of life, performing a meta-analysis proved impractical. Consequently, the findings from these individual studies were presented instead. The NHP scores and EuroQol5D scales showed significant improvement from before to after the intervention. However, the changes in NHP scores (p = 0.1) and EuroQol5D scales (p = 0.935) for RMT did not reach statistical significance between the IG and CG in these studies. Evidence currently available indicates that RMT does not improve quality of life for individuals suffering from chronic stroke.

## 4 Discussion

This study assesses the available evidence for the impact of RMT on respiratory function, exercise capacity, and quality of life in individuals who experienced a chronic stroke. It presents high-quality evidence that RMT can improve primary outcomes, including MIP and respiratory muscle endurance, as well as secondary outcomes like FEV1 and PEF in patients with chronic stroke. Therefore, RMT may be considered a viable therapeutic approach for chronic stroke patients exhibiting these specific metrics. However, current evidence does not indicate that RMT can improve MEP, FVC, exercise capacity, and quality of life in chronic stroke patients, likely attributable to the scarcity of studies available. Thus, more RCTs are needed to study these indicators in future.

The subgroup analysis indicates that IMT + EMT can have benefits on MIP and respiratory muscle endurance, while IMT alone can improve MIP, MEP, and respiratory muscle endurance. This difference may be related to the following factors. While strengthening the inspiratory muscles, IMT alone may indirectly activate the eccentric contraction of the expiratory muscles to maintain chest stability, thereby increasing MEP (Kellens et al., 2011). Furthermore, limited central drive resources after stroke are preferentially allocated to the inspiratory muscles, making MEP gains more dependent on spinal cord neuroplasticity triggered by IMT rather than EMT (Sapienza and Wheeler, 2006). Besides, EMT addition may dilute neuroadaptive focus, the separate expiratory effort competes with IMT-induced neural circuitry remodeling, reducing central motor drive optimization for expiratory muscles (Sapienza and Wheeler, 2006). However, the pooled data indicate that the two different training methods have a statistical difference only in increasing respiratory muscle endurance. Given that only a single study has assessed IMT + EMT within the subgroup analysis, it remains challenging to ascertain which training regimen may more effectively improve respiratory muscle endurance. In light of the limited research on IMT + EMT and IMT, additional RCTs are warranted to further investigate the area of study.

This research demonstrated that RMT could lead to improvements in both MIP and respiratory muscle endurance in chronic stroke patients, which was also seen in IMT + EMT and IMT alone. The MIP improvement value was close to 2 times the MCID, confirming that RMT can substantially enhance the inspiratory force of chronic stroke patients (Menezes et al., 2016). Most previous reviews (Fabero-Garrido et al., 2022; Guillén-Solà et al., 2017; Pozuelo-Carrascosa et al., 2020; Gomes-Neto et al., 2016; Menezes et al., 2018; Wu et al., 2020; Zhang et al., 2020) obtained similar results, although these reviews did not separately observe the effect of RMT on acute and chronic stroke patients. However, the study by Xiao et al. (2012) produced a different result that RMT cannot enhance respiratory muscle strength among patients with stroke. This may be because their study included only two RCTs and did not distinguish between patients with early and chronic stroke. Potential cortico-spinal dysfunction and respiratory centre dysfunction after chronic stroke can cause respiratory muscle weakness and ultimately respiratory dysfunction (Billinger et al., 2012; Guillén-Solà et al., 2017). RMT may improve MIP and respiratory muscle endurance by promoting the recovery of the respiratory center in the cerebral cortex, activating the corticospinal pathway, improving the integration and regulation of respiratory-related sensory input, and enhancing respiratory drive (Liu et al., 2017; Khedr et al., 2000; Jandt et al., 2011). Furthermore, RMT may also induce remodelling and adaptive changes in the respiratory muscles of chronic stroke patients, transforming type II muscle fibres into type I muscle fibres, improving oxygen supply efficiency, and thus improving MIP and respiratory muscle endurance (Li L. et al., 2024; Bonnevie et al., 2015).

Unfortunately, RMT was not found to improve MEP in chronic stroke patients, but it showed a similar trend and approached statistical significance. The finding is different from our previous study (Zhang et al., 2024), which showed that RMT can improve MIP and MEP in early stroke patients. This indicates that the impact of RMT on respiratory muscle strength may vary between early and chronic stroke patients. It may be relevant to the fact that central nervous system plasticity is easier to recover within 3 months and recovers relatively slowly 3 months after stroke onset (Hebert et al., 2016; Langhorne et al., 2011), which means that RMT may be relatively difficult to promote the improvement of respiratory muscles in stroke patients in the chronic stage. The significant increase in MIP and the lack of improvement in MEP reflect phase-specific neuroplasticity in respiratory neural control after stroke (van K et al., 2024). The inspiratory muscles are innervated by bilateral corticospinal tracts and have strong neural compensatory potential, whereas the expiratory muscles rely on the unilateral pyramidal tract, and abdominal muscle recruitment is often impaired after stroke, resulting in low efficiency of expiratory neural drive (van K et al., 2024). RMT may promote neural remodeling by strengthening the central descending pathways of the inspiratory muscles, but has a weaker effect on the reorganization of the expiratory neural circuit (McConnell and Romer, 2004). It may also be connected with the details of RMT. In the chronic phase of stroke, a greater load intensity, appropriate frequency, and duration of RMT may be required to produce more obvious clinical effects. Furthermore, another possibility is that the existing research investigating the impact of RMT on MEP in both early and chronic stroke patients is somewhat scarce. Thus, there remains a necessity for further RCTs to explore the impact of various forms of RMT on MEP in stroke patients across different stages of recovery.

The meta-analysis showed that RMT had a significant effect on FEV1 and PEF but did not affect FVC in chronic stroke patients. The improvement in FEV1 reached the lower limit of MCID, suggesting that although this improvement is statistically significant, additional interventions may be necessary for patients with severe airway obstruction (Chen et al., 2025). Moreover, the improvement in PEF significantly exceeds MCID, indicating that RMT can effectively enhance cough efficacy (Zhang et al., 2022). The results were inconsistent with the previous reviews (Wu et al., 2020; Zhang et al., 2020). These reviews included both acute and chronic stroke patients, whereas our study only included patients with chronic stroke. Subgroup analyses demonstrated that both IMT + EMT and IMT did not yield significant enhancements in FEV1 and FVC. Diaphragmatic dysfunction and respiratory muscle decline occur in chronic stroke patients (Kim et al., 2017), which can lead to reduced lung and chest wall dilation, resulting in chest sclerosis and reduced chest wall compliance (Fabero-Garrido et al., 2022; Estenne et al., 1983; Estenne et al., 1993). This situation may affect lung volume, reduce flow, and lead to restrictive ventilation patterns (Tomczak et al., 2008). RMT may improve pulmonary function by increasing respiratory muscle strength and chest expansion, reducing the weakening of lung tissue elasticity caused by limited activity after stroke (Billinger et al., 2012; Jo and Kim, 2017). Besides, RMT may improve pulmonary function by increasing diaphragmatic movement, promoting venous return, reducing pulmonary blood stasis, and increasing alveolar ventilation and effective gas exchange (Ghannadi et al., 2022; Kim et al., 2014). Notably, this study did not find that RMT could improve FVC in patients with chronic stroke. However, our previous meta-analysis showed that RMT has a positive effect on FVC in early stroke patients (Zhang et al., 2024). These differences may be due to several reasons. Compared with early stroke, the neurological recovery of patients with chronic stroke is relatively stable (Hu et al., 2009; Ripollés et al., 2016), so longer and more intensive RMT may be required to improve respiratory muscle strength, alleviate spasm of the hemiplegic chest wall, increase chest expansion, and improve the elasticity of the lungs and chest cavity, thereby improving FVC. In addition, the study found that RMT could improve MIP in chronic stroke patients, but no improvement in MEP was observed. During the chronic phase, alterations in MIP alone may prove inadequate for improving the elastic recoil properties of lung tissue, consequently influencing variations in FVC. Notably, enhancements in MIP might positively influence FEV1 and PEF in patients suffering from chronic stroke, whereas the absence of improvement in MEP could adversely affect FVC outcomes. An additional factor contributing to these findings may stem from the limited scope of the studies reviewed, as only four focused specifically on the impact of RMT on FVC in chronic stroke individuals.

This review found that RMT could not enhance exercise capacity among chronic stroke patients, which was also shown in IMT + EMT and IMT. However, most of the previous systematic reviews (Fabero-Garrido et al., 2022; Guillén-Solà et al., 2017; Pozuelo-Carrascosa et al., 2020; Menezes et al., 2018; Wu et al., 2020; Zhang et al., 2020) reported the opposite results, which included patients with both acute and chronic stroke. Notably, the finding differs from our previous study results, which suggested that RMT can improve exercise capacity in early stroke patients (Zhang et al., 2024). Rehabilitation of stroke is based on the plasticity of the central nervous system and the reorganization of the cerebral cortex (Pollock et al., 2013). The unique neuroplasticity recovers easily within 3 months after stroke and slows after 3 months (Kim et al., 2017; Langhorne et al., 2011). This means that RMT in the acute phase is more likely to improve cardiorespiratory endurance, and thus promote the exercise capacity of stroke patients. In the chronic phase, stroke patients have a slow recovery in the damaged central nervous system and corticospinal pathways (Hebert et al., 2016). Therefore, RMT may have a relatively slow effect on exercise capacity in patients with chronic stroke. At the stage, insufficient duration and intensity of RMT can also affect this result. Compared with early stroke patients, chronic stroke patients are more likely to have relatively low rehabilitation enthusiasm (Maclean et al., 2000), which may delay the effect of RMT on motor recovery. Besides, limitations of exercise capacity in chronic stroke patients are often multifactorial, and motor impairments may outweigh respiratory limitations, making it difficult to directly attribute improved exercise tolerance to RMT (Cordo et al., 2009). Therefore, the influence of other potential factors on exercise capacity should be considered when interpreting this result, and caution is advised when directly attributing improvements in exercise tolerance to RMT. Although RMT significantly improves MIP, its limited effect on exercise capacity may be related to energy allocation conflicts (McConnell and Romer, 2004). Inefficient respiratory muscles in stroke patients lead to increased ventilation demand during exercise, although RMT strengthens respiratory muscles, it cannot completely eliminate the competitive occupation of cardiac output by respiratory muscles, resulting in insufficient peripheral muscle perfusion (McConnell and Romer, 2004). Simultaneously, central motor drive disorders in chronic stroke patients may limit the conversion of RMT to overall exercise endurance (van K et al., 2024). Additionally, since this review only included high-quality RCTs in chronic stroke patients, the quantity of RCTs included was significantly lower in comparison to previous investigations. Therefore, the lack of adequate RCTs may be another factor.

Research indicates that RMT can enhance respiratory pressures and motor performance in healthy or athletic individuals (Illi et al., 2012; Kowalski et al., 2024). However, our study found no statistically significant effects of RMT on MEP and motor performance in chronic stroke patients. This difference compared to healthy or athletic individuals may be due to the unique neuropathology of stroke. RMT in neurologically intact individuals induces primarily peripheral neural adaptations, such as diaphragmatic hypertrophy and mitochondrial biogenesis (Kowalski et al., 2024; Fabero-Garrido et al., 2022). While corticospinal tract lesions caused by stroke can damage key pathways, manifesting as central motor drive defects leading to reduced autonomic activation of diaphragmatic motor neurons, autonomic nervous system disorders causing cardiopulmonary coupling disorders during exertion, and denervation of the expiratory muscles leading to fibrosis of the abdominal muscles (Jo and Kim, 2017; Fabero-Garrido et al., 2022). Concurrently, diaphragmatic dysfunction and reduced chest wall compliance in chronic stroke patients limit the translation of strength gains into functional ventilation (Teixeira-Salmela et al., 2005; Liu et al., 2017). Furthermore, motor dysfunction like spasticity and balance impairment in chronic stroke patients may attenuate the beneficial effects of RMT on exercise capacity (Lee et al., 2019), whereas the enhanced respiratory function achieved by RMT in unimpaired individuals can directly translate into improved athletic performance (Illi et al., 2012; Kowalski et al., 2024). Therefore, future RMT protocols should prioritize neural re-education while building on structural remodelling.

Taking into account the currently included studies, RMT did not have a significant influence on improving quality of life. As only two studies (Vaz et al., 2021; Britto et al., 2011) focused on quality of life, the result should be interpreted with caution. The study did not find that RMT can improve the exercise capacity of chronic stroke patients. No improvement in exercise capacity can negatively impact the recovery of quality of life in chronic stroke patients (Billinger et al., 2015). It is important to highlight that all studies incorporated in the review had a limited duration of treatment (ranging from 4 to 8 weeks), which may be inadequate for observing meaningful changes in exercise capacity and quality of life among individuals with chronic stroke. This means that there are still some unanswered questions about the true clinical impact of RMT on people with chronic stroke. Additionally, the limited number of studies reporting these variables may cause a lack of evidence. Therefore, there is a pressing need for additional RCTs to explore the impact of various types of RMT on enhancing exercise capacity and quality of life post-stroke.

Current comprehensive evidence appears to endorse the clinical application of RMT in chronic stroke patients, thus it is advisable to implement RMT to facilitate the recovery of respiratory function in this population. Nevertheless, the limitations of the available evidence must be taken into account in clinical application, and the results should be considered with caution. Firstly, the paucity of high-quality trials in this domain resulted in the extraction and synthesis of only nine RCTs, culminating in a limited total sample size, which could introduce a small trial bias. Furthermore, due to the small number of included trials, we were unable to conduct a formal statistical evaluation, so the possibility of publication bias should be considered when interpreting our findings. Although the included studies were heterogeneous and limited in number, we were unable to provide prediction intervals because we used a preregistered RevMan 5.4 analysis framework, which does not have the ability to calculate prediction intervals. This methodological limitation may lead to an underestimation of the true effect dispersion in different clinical settings. Therefore, we recommend that clinical applicability be interpreted with caution, for which we have provided a leave-one-out sensitivity analysis as an alternative approach to assessing variability. These studies were mainly conducted in Korea, Brazil, China, and Turkey. Variations in medical standards, demographic characteristics of participants, and levels of rehabilitation adherence across different nations may further diminish the generalizability of the findings. Eight studies reported the initial MIP and MEP, but the initial indicators varied among different studies. Patients with respiratory muscle weakness usually may have better effects (Zhang et al., 2024). These situations should be taken into consideration in clinical applications. Additionally, the included RCTs employed a variety of RMT regimens with respect to training load, length, and device, resulting in substantial heterogeneity between interventions, which limited our ability to derive clinically actionable recommendations. Most of the studies implemented RMT as an adjunct to conventional rehabilitation, and the observed improvements in overall functional levels may have led to an overlap in treatment effects, thereby impacting the validity of the outcomes. Future studies should establish optimal RMT protocols using sham-controlled RCTs to quantify true dose-response relationships regarding frequency thresholds (e.g., ≥5 sessions/week), minimum effective duration (e.g., 6–8 weeks vs longer), and intensity progression models (e.g., 30%–80% MIP) for chronic stroke populations. Furthermore, while this study focused on the effects of strength-based RMT on patients with chronic stroke, future studies should explore other respiratory muscle training methods, such as tapered flow resistance training or VIH, which have shown promise in other populations but remain understudied in the treatment of chronic stroke.

Although respiratory muscle endurance demonstrated significant improvement, the clinical significance of this enhancement requires evaluation against individualized functional goals due to the absence of MCID thresholds specific to respiratory muscle endurance in chronic stroke patients. Future studies should establish a specific MCID threshold for chronic stroke patients to guide clinical practice. The lack of inclusion of CPET measures in existing trials is a significant limitation. Though CPET provides the most comprehensive assessment of exercise capacity, it is not frequently used in current studies of RMT in chronic stroke, forcing reliance on secondary outcomes such as 6MWT. While 6MWT is a validated and widely used measure in stroke rehabilitation, it cannot isolate respiratory-specific improvements, which may mask the benefits of RMT on exercise capacity. This inherent limitation stems from the fact that the 6MWT integrates systemic factors such as lower limb motor compensation and cardiovascular adaptations. Thus, Future trials should prioritize CPET to assess the effects on motor function in this population. Moreover, not all reported the same variables, and some variables (like PEF and quality of life) were only analysed in 2 or 3 studies. Regrettably, the inability to perform a meta-analysis on quality of life outcomes is due to the variability in assessment tools used in the 2 studies. Consequently, the apparent lack of improvement in quality of life attributed to RMT may reflect insufficient evidence rather than a true absence of efficacy. Future trials should prioritize quality of life as a primary endpoint using standardized, validated instruments to conclusively determine the impact of RMT on chronic stroke patients. Additionally, results from subgroup analyses also should be considered with caution, as some measures are based on only 1 or 2 studies. This may lead to unstable estimates, increased variability, and reduced statistical power, which may compromise the reliability of the findings. Furthermore, the study primarily focused on outcomes including respiratory function, exercise capacity, and quality of life during the design phase, while several other clinically significant indicators, including respiratory complications, pneumonia, therapeutic effects on cough, or recurrence of respiratory failure, were excluded. RMT may improve these variables in chronic-phase stroke patients. Therefore, future research will further investigate whether RMT has an impact on these indicators in chronic stroke patients. Ultimately, considering sex differences in the respiratory system, women have smaller lung volumes, reduced alveolar diffusion surface areas, and relatively narrower airway diameters compared with men of the same height and age (Sheel and Guenette, 2008; Hopkins and Harms, 2004). Therefore, women demonstrate increased work of breathing, higher airway hyperresponsiveness, expiratory flow limitation, and potentially greater exercise-induced arterial hypoxemia compared with men (Guenette et al., 2007). Notably, sex-based differences in RMT effects have been observed, particularly concerning adverse symptoms such as headache and dizziness, which are more pronounced in females (Kowalski et al., 2023). This may be attributed to greater blood gasometry changes (e.g., hypercapnia) leading to increased cerebral blood flow and elevated intracranial pressure (Rodriguez et al., 1988; Ainslie and Duffin, 2009; Ramadan, 1996). Therefore, safety and feasibility must be prioritized in vulnerable populations, including chronic stroke patients, to mitigate these risks and ensure effective implementation of RMT interventions. Although the number of men and women included in this study was relatively balanced (143 men and 145 women), the original study lacked sex-stratified outcome data. We just used the percentage of women at baseline for subgroup analysis. Future research should prioritize sex-stratified clinical trials to explore whether sex differences significantly affect the application of RMT in chronic stroke patients.

## 5 Conclusion

This review provides robust evidence that RMT significantly enhances primary outcomes, including MIP and respiratory muscle endurance, as well as secondary outcomes like FEV1 and PEF in individuals who experienced a chronic stroke. However, no evidence of effect has been found for RMT on improving MEP, FVC, exercise capacity, and quality of life in this patient population. IMT + EMT, as well as IMT alone, can enhance MIP and respiratory muscle endurance. Furthermore, IMT alone can improve MEP. More well-designed RCTs with multi-center, randomized, double-blind, and placebo training are required to investigate the optimal RMT for individuals who experienced chronic strokes, including modality, intensity, frequency, and duration.

## Data Availability

The original contributions presented in the study are included in the article/supplementary material, further inquiries can be directed to the corresponding author.

## Abbreviations

RCTs, Randomised controlled trials; PEDro, The physical therapy evidence database; PICOS, Population-Interventions-Comparison-Outcomes of interest-Study design; IG, Intervention Group; CG, Control Group; RMT, Respiratory muscle training; IMT, Inspiratory muscle training; EMT, Expiratory muscle training; VC, Vital capacity; MIP, Maximal Inspiratory Pressure; MEP, Maximal Expiratory Pressure; RME, Respiratory muscle endurance; FEV1, Forced Expiratory Volume in 1 s; PEF, Peak Expiratory Flow; FVC, Forced Vital Capacity; 6MWT, Six-minute walk test; MAS, Maximum activity score; EQ5D, euroquol 5-dimensions; NHP, Nottingham health profile; MD, Mean difference; SMD, Standardized mean difference; 95% CI, 95% Confidence intervals; JBI, The Joanna Briggs Institute; NR, Not reported in the source trial; N/A, Not available
